# Gut Microbiota Dysbiosis Is a Key Driver of Inflammaging in Chronic Kidney Disease

**DOI:** 10.3390/cells15131171

**Published:** 2026-06-27

**Authors:** Emanuele Parodi, Luigi Mario Castello, Paolo Bottino, Franca Gotta, Marialuisa Novi, Marco Orsello, Andrea Rocchetti, Stefania Prenna, Vincenzo Cantaluppi, Marco Quaglia

**Affiliations:** 1 Nephrology and Dialysis Unit, SS. Antonio e Biagio e Cesare Arrigo University Hospital, 15121 Alessandria, Italy; emanuele.parodi@ospedale.al.it; 2 Internal Medicine Unit, SS. Antonio e Biagio e Cesare Arrigo University Hospital, 15121 Alessandria, Italy; luigi.castello@ospedale.al.it; 3 Department of Translational Medicine, Università del Piemonte Orientale (UPO), 28100 Novara, Italy; 20022114@studenti.uniupo.it (S.P.); vincenzo.cantaluppi@med.uniupo.it (V.C.); 4 Microbiology Unit, SS. Antonio e Biagio e Cesare Arrigo University Hospital, 15121 Alessandria, Italy; paolo.bottino@ospedale.al.it (P.B.); fgotta@ospedale.al.it (F.G.); arocchetti@ospedale.al.it (A.R.); 5 Gastroenterology Unit, SS. Antonio e Biagio e Cesare Arrigo University Hospital, 15121 Alessandria, Italy; mnovi@ospedale.al.it (M.N.); marco.orsello@ospedale.al.it (M.O.); 6 Nephrology and Transplant Unit, Maggiore della Carita University Hospital, 28100 Novara, Italy

**Keywords:** gut microbiota, dysbiosis, chronic inflammation, inflammaging, frailty, chronic kidney disease, end-stage kidney disease

## Abstract

The role of gut microbiota and intestinal dysbiosis in promoting inflammaging in chronic kidney disease (CKD) has been the focus of intense research over the last years. Some alterations at the phyla level, such as abundance of *Proteobacteria* and reduction in *Firmicutes/Bacteroidites* (F/B) ratio and saccarolytic populations, have been consistently reported in CKD. Other mechanisms include microbial translocation through a “leaky gut” and subsequent molecular mimicry, immune dysregulation (unbalance between T reg and Th17 subsets), and epigenetic interactions. Alterations of metabolic pathways and of bacterial metabolites, such as butyrate and other short chain fatty acids (SCFA), also appear to play a key role in modulating progression of CKD. On the other hand, microbiota-based therapy appears promising and includes diet, prebiotics, probiotics, synbiotics, postbiotics and fecal microbiota transplantation (FMT). Modulation of microbiota could correct critical alterations, such as F/B ratio and T reg/Th17 unbalance, blunting inflammaging and potentially reducing progression of CKD and cardiovascular disease. Despite current limitations, gut microbiota is emerging as a powerful environmental factor which could be harnessed to interfere with key mechanisms leading to inflammaging in CKD.

## 1. Introduction

Chronic kidney disease (CKD) globally affects 10–15% of adults, is increasing over recent decades, and has significant implications for morbidity and mortality [1]. A hallmark of CKD is the presence of a chronic, low-grade inflammation characterized by biological pathways associated with aging, termed “inflammaging” [2]. This process is shared by many age-related disorders and contributes to the development of vascular calcifications [3] and the disproportionate increase in cardiovascular risk in CKD compared to the general population [4]. Inflammaging is closely linked to many other peculiar aspects of frailty of CKD [5], such as sarcopenia, osteoporosis [6] and cognitive decline [7].

Etiopathogenesis of inflammaging is multifactorial and includes immunosenescence [8], mitochondrial dysfunction [9], persistent exposure to bacterial antigens and chronic activation of the innate immune system [10]. Building evidence, especially within the setting of obesity, also suggests that gut microbiota (GM) alterations, or dysbiosis, can fuel this process by impairing the intestinal function of gatekeeper of inflammatory stimuli [11,12,13]. Consistently, gut microbiota manipulation seems to represent a new tool to blunt inflammaging and consequently reduce cardiovascular risk and frailty in CKD populations [14].

The main aim of the review is to provide a state-of-the-art analysis of the role of gut microbiota in promoting inflammaging in CKD and of gut microbiota manipulation in blunting this process. The PubMed library was searched from inception to December 2025, using a combination of Medical Subject Headings (MeSH) and keywords related to gut microbiota, dysbiosis, chronic inflammation inflammaging, frailty, chronic kidney disease, and end-stage kidney disease.

## 2. Physiological Functions and Immunological Role of Gut Microbiota

Gut microbiota exert many important physiological functions, ranging from modulation of lipid and glucose metabolism to regulation of the immune system (Table 1), and interact with many organs including the heart, kidney, muscles and central nervous system [15].

Interaction with the immune system plays a multifaceted role. Gut microbiota help maintain tolerance towards commensal-derived antigens which are constantly sensed by gut-associated lymphoid tissue (GALT), preventing development of chronic inflammatory conditions like inflammatory bowel disease (IBD) and systemic autoimmune disorders. Induction of commensal-specific anti-inflammatory CD4^+^ T-cell responses, especially Foxp3^+^ regulatory T cells (Foxp3^+^ Tregs) and Type 1 regulatory T cells (Tr1), is key in this process, as both of them can induce the release of anti-inflammatory cytokine IL-10. Recent evidence also underlines the role of group 3 innate lymphoid cells (ILC3s)-intrinsic STING signaling in the instruction of microbiota-specific Tregs at steady state. However, heightened STING activation due to gut inflammation becomes detrimental because it results in the death of tissue-protective ILC3 [27]. In addition, even effector T-cell subsets, such as Th17 cells, can acquire an anti-inflammatory profile within the intestine. All these tolerogenic processes are modulated by specific bacterial species (Lactobacillus and Bacteroides genera) and microbial-derived structural components and metabolites, which also induce secretion of anti-inflammatory mediators by intestinal epithelial cells, dendritic cells (DCs) and macrophages [28].

A disrupted balance between these signals may result in low-grade chronic inflammation and inflammaging [21].

Many environmental factors can influence gut microbiota and alter their balance. The main ones include early life, chronic psychological stress, sleep and circadian rhythm, smoking, exercise, drugs, gastrointestinal infections, pollution, lifestyle (rural vs. urban) and diet [29]. Interestingly, vitamin D plays an important role in preserving gut barrier integrity [30], regulating microbial diversity and modulating immune responses [31].

## 3. Dysbiosis and Gut–Kidney Axis in Chronic Kidney Disease (CKD)

### 3.1. Causes of Dysbiosis in CKD

CKD is associated with many factors predisposing to alterations of gut microbiota, including possible intestinal ischemia, constipation due to poor fiber intake, mainly due to restriction of potassium-rich fruits and vegetables, metabolic acidosis, administration of phosphate and potassium chelating agents and iron supplementation, retention of uremic toxins and increase in urea circulating levels, with consequently increased urea concentration in the intestinal lumen and expansion of the urease-positive bacterial population. The latter alteration results in an increased production of ammonia and increased luminal pH, which contributes to leaky gut syndrome and further worsens dysbiosis [32].

A protein-rich diet and uremic conditions can lead to the overgrowth of protein-fermenting bacteria, producing toxic byproducts like indoxyl sulfate (IS), p-cresyl sulfate (p-CS), and ammonia. These protein fermentation metabolites contribute to systemic toxicity, promoting the progression of CKD and associated cardiovascular complications [33].

A metabolomic study on end-stage renal disease (ESRD) patients by Vaziri et al. documented an expansion of bacterial families expressing urease and uricase and indole and p-cresol-forming enzymes, along with a reduction of those with butyrate-forming enzymes, and a global shift towards proteolytic mechanisms [34].

### 3.2. Metabolic Consequences of Dysbiosis in CKD: Accumulation of Gut-Derived Uremic Toxins and Depletion of Short-Chain Fatty Acids

Dysbiosis promotes intestinal production of several uremic toxins, including protein-bound uremic toxins (PBUT) such as IS, p-CS and indole acetic acid (IAA), and water-soluble toxins such as trimethylamine N-oxide (TMAO) and phenylacetylglutamine (PAG). These toxins are collectively referred to as “gut-derived uremic toxins” (GDUT) and exert a significant impact on both cardiovascular risk and CKD progression [35].

Gut microbiota dysbiosis has been actually implicated in endothelial dysfunction, arterial hypertension, atherosclerosis, heart failure and myocardial infarction through mechanisms mediated by increased production of gut-derived uremic toxins and decreased availability of short-chain fatty acids (SCFA) [36].

The main effects of gut-derived uremic toxins on mechanisms of inflammaging and fibrosis are outlined in Table 2.

Dysbiosis contributes to increased circulating levels, and CKD amplifies this effect through impaired kidney excretion, especially when GFR falls below 60 mL/min. Protein-bound uremic toxins play a crucial role in endothelial dysfunction and are not easily removed by dialysis as they are highly bound to plasma proteins [50]. They exert vascular and renal toxicity through multiple mechanisms [49]. We will briefly analyze the potential clinical impact of main gut-derived uremic toxins.

IS can determine endothelial dysfunction, and higher serum total levels were associated with increased all-cause mortality and infectious events in Japanese dialysis patients [37] but not in other studies [40] including HEMO study [51]. Free levels of IS and p-CS predicted development of heart failure in non-dialyzed CKD patients [38]. Detrimental mechanisms of actions of IS are detailed in Table 2.

p-CS has a wide range of toxic effects on cardiovascular system, and a meta-analysis study showed that elevated free levels correlate with increased all-cause mortality and risk of CV events in CKD [40]; similarly, free p-CS showed an independent association with CV events (fatal and nonfatal) in a cohort of 523 patients with non-dialysis CKD at stages 1–5 [41]. Serum p-CS was also independently associated with aortic stiffness and vascular calcifications in the same setting [42] (Table 2).

Increased IAA levels have been associated with higher thrombotic risk and both cardiovascular and all-cause mortality in CKD patients [41] and with heart hypertrophy and oxidative stress in cardiac tissues in animal models [43]. In addition, they appear to correlate with monocyte-to-high-density lipoprotein ratio, an emerging biomarker of cardiovascular disease [52] (Table 2).

TMAO is a prominent driver of atherosclerosis [45], and elevated levels have been associated with a 55% higher risk of all-cause mortality in hemodialysis patients in a meta-analysis [46], a trend confirmed in recent studies [47]. It contributes to cardiovascular disease by promoting inflammation, foam cell formation, arterial cholesterol deposition and platelet hyperreactivity [53] (Table 2).

PAG acts on G-protein-coupled receptors, promoting platelet responsiveness, thrombosis and atherosclerosis [48,49] (Table 2).

Overall, despite discrepancies, available evidence suggests that all gut-derived uremic toxins can contribute to inflammaging and that their levels are associated with mortality and cardiovascular outcomes, especially at more advanced stages of CKD [35].

In addition to accumulation of gut-derived uremic toxins, CKD is also characterized by reduced levels of circulating SCFA, such as butyrate, acetate and propionate, as compared to healthy controls. This depletion is inversely correlated with CKD stage and is especially severe in hemodialysis patients [54]. SCFA exert a wide range of beneficial effects: they improve gut barrier integrity and function, modulate blood pressure and glucose and lipid metabolism [55], promote immunotolerance by expanding Foxp3^+^ regulatory T cells [56] and blunt systemic and renal inflammation in CKD, reducing interstitial fibrosis [57] and protecting the integrity of the glomerular barrier [58]. Thus, a reduction in SCFA-producing bacteria can worsen inflammation and contribute to the progression of CKD [59,60].

Defining the role of dysbiosis and diet in modulating their levels in CKD needs further investigation as it could pave the way for new approaches to reduce the levels of uremic toxins [61].

## 4. The Link Between Dysbiosis and Inflammaging in CKD

Dysbiosis has a remarkable impact on increased cardiovascular risk which characterizes CKD. This is mediated by the production of protein-bound uremic toxins, modulation of nitric oxide (NO) and SCFA availability [62].

**Figure 1:**
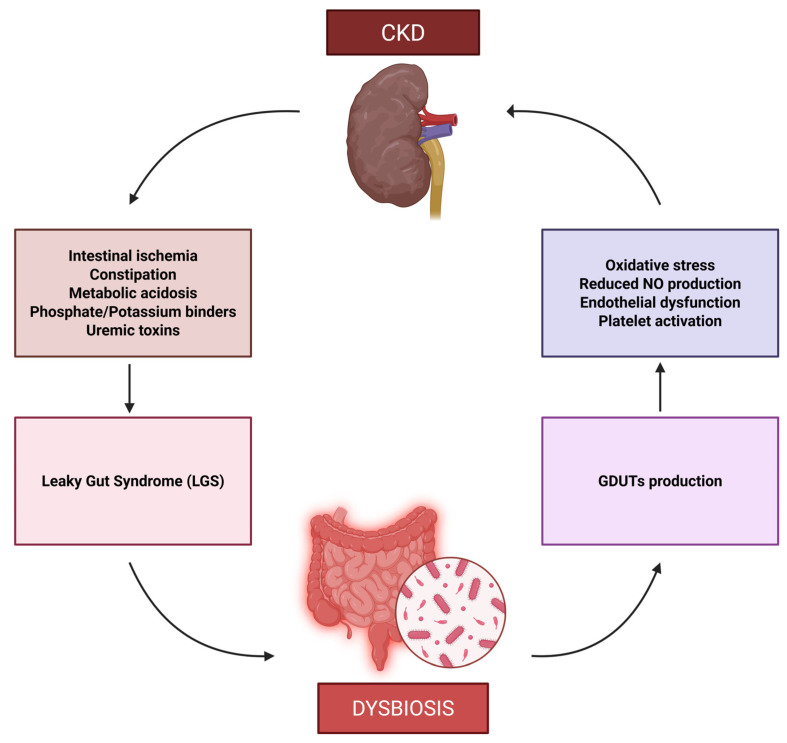
Vicious circle between CKD and intestinal dysbiosis. Several CKD-related factors predispose one to LGS and intestinal dysbiosis (left-hand arrows) whereas intestinal dysbiosis determines production of GDUT which promotes progression of CKD through oxidative stress and other mechanisms (right-hand arrows). Accumulation of circulating GDUT due to progressive reduction of renal function further worsens intestinal dysbiosis, creating a vicious circle. Abbreviations: CKD: chronic kidney disease; GDUT: gut-derived uremic toxins; LGS: leaky gut syndrome; NO: nitrogen oxide.

These toxins have pro-oxidative, pro-senescence, pro-inflammatory and pro-fibrotic effects which cause systemic and renal inflammation, accelerating renal fibrosis and CKD progression [63]. This creates a vicious circle of chronic vascular and renal inflammation, progression of CKD and of cardiovascular disease, further accumulation of protein-bound uremic toxins and other uremic toxins, and worsening of gut microbiota dysbiosis and leaky gut syndrome (Figure 1).

Endotoxemia due to leaky gut syndrome, immune activation, oxidative stress and DNA damage are the main mechanisms linking dysbiosis to inflammaging in the setting of CKD [64]. This state of low-grade chronic inflammation is characterized by a downregulation of the innate immune system, an upregulation of pro-inflammatory cytokines (IL-6, IL-18 and TNF-α) and activation of the NF-κB signaling pathway [65].

Alteration of IFNγ levels may also contribute to this setting by amplifying mucosal barrier dysfunction and skewing the immune system towards a Th1 response [32].

Accumulation of senescent cells in different tissues, including adipose, heart and kidney, maintains this condition due to a hypersecretory phenotype, “senescence-associated secretory phenotype” (SASP) [66].

**Figure 2:**
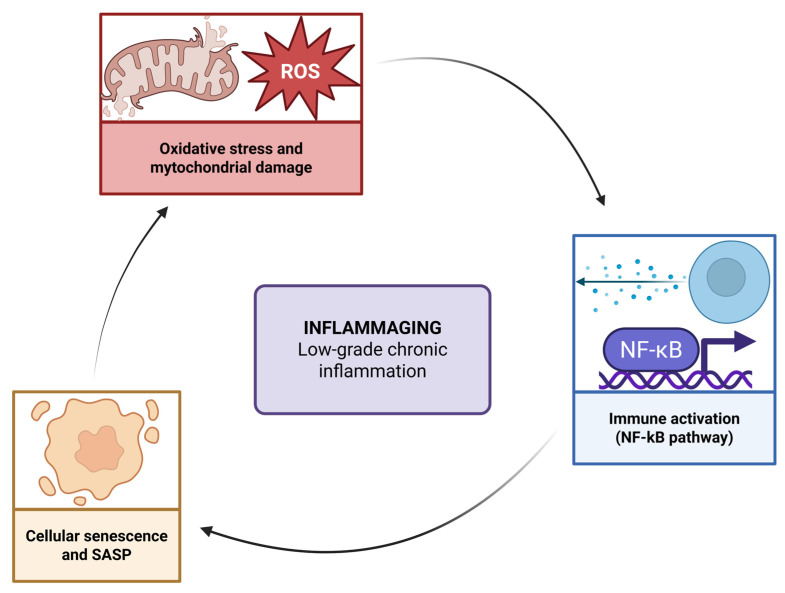
Vicious circle connecting different drivers of inflammaging: mitochondrial oxidative stress, activation of NF-kB and other inflammatory pathways, promotion of cellular senescence and SASP phenotype. Abbreviations: NF-kB: nuclear factor kappa-light-chain-enhancer of activated B cells; SASP: senescence-associated secretory phenotype.

Oxidative stress triggered by intestinal dysbiosis and translocation of uremic toxins is closely linked to inflammaging as it can directly damage mitochondrial DNA (mtDNA), which is highly prone to damage from reactive oxygen species (ROS) due to limited repair capacity. Oxidative damage can increase mtDNA mutations, impairing mitochondrial respiratory chain and favoring release of mitochondrial ROS (mtROS), which further contribute to cellular senescence and inflammaging [67]. This self-amplifying mechanism is described in Figure 2.

Recent evidence suggest that mechanisms of epigenetic regulation such as DNA methylation, histone modifications and non-coding RNA interactions are involved in the fine-tuning of the mitochondrial quality control system and can promote pathogenesis and progression of diabetic kidney disease [68].

Reduced availability of SCFAs due to intestinal dysbiosis can impair this epigenetic regulation, determining overexpression of fibrosis-related genes and promoting chronic renal damage and accelerated renal aging through shared mechanisms [69].

### 4.1. Clinical Manifestations Associated with Dysbiosis and Inflammaging in CKD

Dysbiosis has been associated with several features of uremia. We will analyze the most significant associations.

#### 4.1.1. Cardiovascular Manifestations

Metabolites released by gut microbiota have an impact on non-traditional cardiovascular risk factors of CKD, such as chronic inflammation and endothelial dysfunction [70]. A hallmark of uremic vasculopathy is represented by vascular calcifications [71]. Gut dysbiosis is associated with vitamin K insufficiency due to a decreased population of vitamin K-producing bacteria, leading to oxidative stress, inflammation and progression of vascular calcifications in hemodialysis patients [72]. Vitamin K plays a crucial role in inhibiting the process of vascular calcification through several vitamin K-dependent proteins such as matrix Gla protein [73]. A strong association between insufficient vitamin K levels, inactive matrix Gla protein, and increased CVD risk has been demonstrated across CKD stages [74].

Gut microbiota also play an important role in arterial hypertension. An altered crosstalk between the gut, the kidney and the brain (brain–gut–kidney axis) can predispose to hypertension in CKD. Interestingly, a diet rich in salt can enhance expansion of Th 17 cells, thus potentiating a key inflammatory mechanism of dysbiosis [75].

An association between gut microbiota dysbiosis and brain vascular damage has been demonstrated in experimental models of aging and CKD, with a correlation between TMAO and IS and cerebral microhemorrhages [76].

Dysbiosis probably plays an important role in mediating the causal relationship between CKD and cognitive dysfunction, and a Mendelian randomization study indicated specific gut microbes, such as the *Eubacterium fissicatena* group, as potential modulators of cognitive decline [77].

Metabolites such as IS have also been associated with CKD-related heart failure. Interestingly, IS can disrupt cardiac mitochondrial function and induce myocardial apoptosis. As it is produced by *E. coli* through the tryptophanase, administration of probiotics which can reduce *E. coli* abundance and IS levels is promising and has resulted in improved cardiac outcomes in rats and patients with CKD [78].

Dysbiosis of gut microbiota could also contribute to uremic cardiomyopathy through induction of IFNγ-producing CD4^+^ T cells, which can infiltrate the heart and correlate with diastolic dysfunction. Marked expansion of intestinal opportunistic pathogens, particularly Klebsiella pneumoniae, is observed in CKD and appears to trigger this process in experimental models of uremic cardiomyopathy [79].

#### 4.1.2. Sarcopenia

This is another important CKD-associated clinical manifestation. It has a multifactorial etiopathogenesis mainly including uremia, hyperparathyroidism, and low-grade systemic inflammation driven by gut microbiota dysbiosis [80].

#### 4.1.3. Malnutrition

Finally, gut microbiota is important for maintaining the nutritional status of patients. In a study on hemodialysis patients, categorized according to Geriatric Nutritional Risk Index (GNRI), genus Bifidobacterium was less represented in the high-GNRI group than in the low-GNRI one. At the species level, deficiency of *B. adolescentis* and *B. bifidum* was strongly associated with an increased nutritional risk. Interestingly, Bifidobacterium levels inversely correlate with IL-6 and TNFα levels, suggesting that modulation of chronic inflammation may mediate effects on nutritional status [81].

## 5. Modulation of Gut Microbiota in CKD

**Figure 3:**
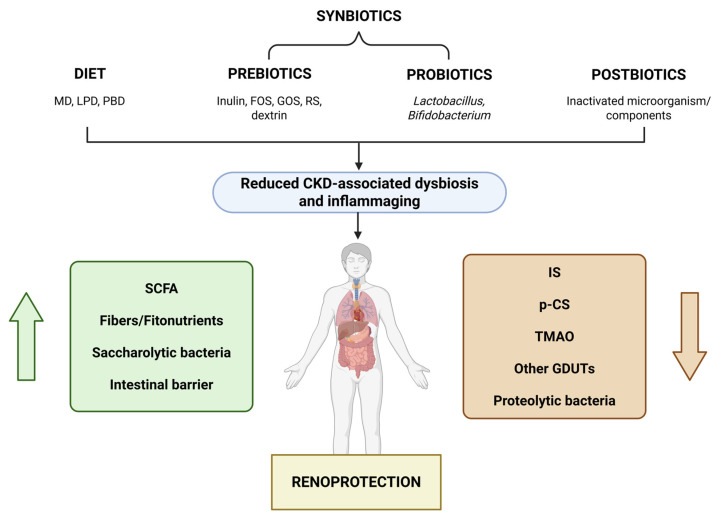
An integrated therapeutic approach with diet and biotics can reduce inflammaging through multiple mechanisms and exert a nephroprotective effect. Abbreviations: CKD Chronic kidney disease; FOS: fructo-oligosaccharides; GDUT: gut-derived uremic toxins; GOS: galacto-oligosaccharides; IS: indoxyl sulfate; LPD: low protein diet; MD: Mediterranean diet; PBD: plant-based diet; p-CS: p-cresyl sulfate; RS: resistant starch; SCFA: short-chain fatty acids; TMAO: trimethylamine N-oxide.

Dietary interventions, administration of different types of biotics and fecal microbiota transplantation (FMT) are being investigated for their ability to correct dysbiosis, with the rationale of strengthening the gut barrier and lowering uremic toxin loads in CKD, consequently reducing inflammaging and the risk of cardiovascular events and CKD progression. An integrated approach combining these tools and its potential beneficial effects are outlined in Figure 3.

### 5.1. Diet

Dietary interventions play an important role in modulating inflammaging in patients with CKD. A low-protein diet has been reported to decrease p-CS production in some studies, as the precursors for p-CS and other gut-derived uremic toxins are derived from protein fermentation in the gut [82]. However, a meta-analysis published in 2021 did not indicate any significant changes in IS and p-CS levels, nor any difference in renal function, between CKD patients who started with a low-protein diet and those who continued on a normal diet. The impact of a low-protein diet on gut microbiota occurred predominantly at the families and species levels (enrichments of *Lactobacillaceae*, *Bacteroidaceae* and *Streptococcus anginosus*) but was minimal on microbial diversity and did not appear to alter clinical outputs [83]. A recent study on a murine model of DKD, however, suggested that a low-protein, calorie-restricted diet blunted gut microbiota dysbiosis, corrected increased Firmicutes/Bacteroidetes ratio and reduced serum TMAO levels. Suppression of NOD-like receptor family pyrin domain containing 3 (NLRP3) and interleukin-1β (IL-1β) at the tissue level was also demonstrated, resulting in mitigated kidney damage [84]. The reason for these inconsistent results may lie in differences in species, study design, dietary composition and outcome measures, which limit the power of meta-analysis and comparison between studies.

Overall, more studies are needed to explore the clinical impact of a low-protein diet in humans with CKD and elucidate underlying mechanisms of nephroprotection which have been emerging in animal models [85].

Recent studies have underlined the value of plant-based diets in modulating gut microbiota and improving metabolic health in CKD [86], with possible beneficial effect on cardiovascular risk [87].

Plant-based diets include two core components, both of which are active in modulating gut microbiota: dietary fibers and phytochemicals. Dietary fibers can be subdivided into non-starch polysaccharide (e.g., cellulose, inulin), resistant oligosaccharide (e.g., α-galacto-oligosaccharides or GOS, β-fructooligosaccharides or FOS), resistant starch (RS) and other fibers [88]. Their role as prebiotics in CKD is important and will be discussed in the following section.

Phytochemicals are non-nutrient compounds with anti-inflammatory and antioxidant properties. For example, plasma levels of linoleic acid inversely correlated with IL-6 levels and all-cause mortality in a Swedish study of dialysis patients [89]; polyphenols modulate gut microbiota and affect a wide range of key signaling pathways involved in inflammaging, such as nuclear factor kappa-B (NF-κB), mitogen-activated protein kinase (MAPK) and mammalian target of rapamycin (mTOR) [90].

Long-term plant-based diets can achieve significantly lower production of p-CS and IS and reduce the risk of CKD progression and death. In a study on elderly patients with stage 3–5 CKD, a high adherence to this diet, assessed with a plant-based diet index, was independently associated with higher insulin sensitivity and lower levels of IL-6 and C-reactive protein [91].

The Mediterranean diet, characterized by vegetable intake several times a day and reduced intake of gut-derived uremic toxin precursors such as phenylalanine and tyrosine, has also been associated with lower p-CS concentrations among hemodialysis and peritoneal dialysis patients and even in kidney transplant recipients, with a more pronounced effect in those with higher adherence. This study underlies the association between nutrition and circulating gut-derived uremic toxins across different types of kidney replacement therapy [92].

Unlike a Western diet, which favors the growth of proteolytic bacteria, increased production of protein-bound uremic toxins and cardiovascular and renal aging [93], the Mediterranean diet promotes the proliferation of saccharolytic populations, due to its high content of fermentable carbohydrates along with vegetable proteins and fish oil.

Fermented foods (such as Kefir, kimchi and yoghurt) can also play a role in blunting inflammaging, and their regular consumption in early stages of CKD could prevent progression of kidney damage [94]. A secondary analysis of NHANES showed that frequent use (≥3 weekly) of yoghurt, a rich natural probiotic source, was associated with decreased risk for onset of proteinuria [95]. Higher yoghurt and milk consumption was recently associated with a lower likelihood of developing CKD and metabolic syndrome and with a better control of cardiovascular risk factors in a large Japanese population with type 2 diabetes [96]. Similarly, daily intake of fermented vegetables was associated with a reduced incidence of CKD in 9.229 subjects with cardiometabolic disease and normal kidney function [97].

In addition to diet, a combined intervention of a low-protein diet and different types of biotics has been investigated and will be analyzed in the following section.

### 5.2. Biotics

#### 5.2.1. Prebiotics

Prebiotics are indigestible food components which promote expansion of beneficial intestinal bacteria [88]. They include fibers, such as inulin, FOS, GOS, RS and dextrin, which ferment in the gut to produce SCFAs [98]. The results of the main studies of these types of prebiotics are summarized in Table 3.

Fermentable dietary fibers, carbohydrates which cannot be completely digested, play a key role in modulating gut microbiota because they promote expansion of SCFA-forming saccharolytic bacterial populations, limiting proteolytic bacteria which produce putrefactive metabolites such as gut-derived uremic toxins. Dietary restriction of potassium-rich fruits and vegetables in advanced CKD stages results in a reduced intake of fibers, which favors this mechanism of dysbiosis [105].

Dietary fiber inulin and inulin-type prebiotics can increase the abundance of *Bifidobacterium and Faecalibacterium prausnitzii*, both characterized by anti-inflammatory properties; in addition, inulin and its intestinal metabolites exert direct immunomodulatory roles promoting expansion of Tregs [106].

Inulin supplementation also appears to delay CKD-mineral and bone disease in rats with CKD, lowering phosphorus and parathyroid hormone levels and blunting both bone remodeling and cardiovascular calcifications [107].

When started in association with a low-protein diet in CKD patients, inulin also determined a significant improvement in glucose and lipid metabolism and CRP [108]. In a more recent study, the addition of inulin, calcium citrate and α-chetoanalogues to a low-protein diet improved eGFR and uremic symptoms in advanced CKD [100].

In the TarGut-CKD Study, a nonrandomized, open-label, pilot trial, oligofructose-enriched inulin determined an expansion in saccharolysis-derived metabolites and an abundance of *Bifidobacterium and Anaerostipes* [99].

RS promotes proliferation of Bifidobacterium and Lactobacillus and can increase the production of SCFA and other beneficial metabolites [101]. A meta-analysis indicated that RS can reduce circulating IS and urea levels and help preserve renal function in CKD [101], and a recent RCT demonstrated its positive effect on p-CS levels and a reduction of Bacteroides [102].

GOS (present in legumes such as lentils, dairy products and some root vegetables) can limit pathogenic bacteria and enhance production of anti-inflammatory cytokines such as IL 10 in models of neuroinflammation and CKD [109].

A randomized, placebo-controlled, double-blind, cross-over study compared prebiotic arabinoxylan oligosaccharide (AXOS) with maltodextrin for 4 weeks and detected no effect on gut-derived uremic toxins and insulin resistance in CKD patients [103]; however, resistant maltodextrin had a beneficial impact on intestinal occluding and zonula occluden-1 up-regulation in another study, in which it promoted combined abundance of *Lactobacillus*, *Bifidobacteria*, *Akkermansia*, and *Roseburia* in CKD rats [110].

Associating prebiotics and probiotics as synbiotics may be more effective in modulating gut microbiota, and synbiotic formulations are thought to enhance the survival and colonization of probiotics by providing a nutritional source (prebiotics) [111]. Synbiotics will be analyzed in Section 5.2.3.

#### 5.2.2. Probiotics

In an open-label, placebo-controlled trial, a 3-month intervention with Lactobacillales and Bifidobacteria improved iron kinetics, inflammatory parameters, and lipid metabolism but had no significant effect on eGFR) [112].

Similarly, a 12-week probiotic supplementation using *Lactobacillus acidophilus*, *Lactobacillus casei*, and *Bifidobacterium bifidum* in diabetic patients undergoing hemodialysis improved glucose homeostasis parameters and certain biomarkers of inflammation and oxidative stress and the Subjective Global Assessment (SGA) score, an indicator of nutritional status, whereas it did not significantly affect kidney function [113].

The ProLowCKD study evaluated the addition of probiotics to a low-protein diet (0.6 g/kg body weight) for 3 months in patients with CKD and an eGFR < 25 mL/min. Administration of the probiotic was associated with a lower need for diuretic and antihypertensive drugs and lower levels of urinary toxins [114].

In a RCT, 48 diabetic patients with DKD were assigned to a diet containing probiotic-enriched soy milk (200 mL/d) or soy milk in the control condition. A panel of biomarkers of oxidative stress was measured after 8 weeks of intervention and showed that patients treated with probiotic soy milk had lower levels of oxidative stress [115].

Oral administration of the probiotic *Lactobacillus casei Zhang* (L. casei Zhang) corrected gut microbial dysbiosis in a murine model of bilateral renal ischemia-reperfusion (I/R)-induced, blunting kidney injury and slowing progression to CKD. Interestingly, *L. casei Zhang* was proven to elevate SCFAs and nicotinamide (NAD) levels both in the serum and within the kidney, increasing expression of enzymes involved in NAD synthesis and of gene coding for Peroxisome proliferator-activated receptor gamma coactivator 1-alpha (PGC1α), a key regulator of mitochondrial biogenesis. These mechanisms determined an anti-oxidant and anti-inflammatory effect and reduced renal tubular epithelial cell damage and interstitial fibrosis; of note, these effects could be transferred with stool transplant [116]. On this basis, the same group performed a phase 1, placebo-controlled study of oral *L. casei Zhang* use (Chinese clinical trial registry, ChiCTR-INR-17013952), which confirmed in a clinical setting that this probiotic can blunt proteinuria and slow the decline of kidney function in stage 3–5 CKD after a follow-up of 10 months. Interestingly, even the pasteurized forms of *Lactobacillus casei Zhang* maintained protective effects on AKI and chronic renal fibrosis, suggesting other mechanisms beyond the interaction of live probiotics with the host [117].

In a recent RCT, *Lacticaseibacillus rhamnosus* L34 (L34) was tested and compared with *L. rhamnosus GG* (LGG) and placebo in patients with stage 3–5 CKD. A 4-week course of L34 was initially tested in a pre–post study and showed a reduction in gut derived uremic toxins, except IS, and attenuation of several biomarkers related to inflammation (including neutrophil extracellular traps, cell-free DNA) and altered gut permeability (beta-D-glucan) and of gut dysbiosis. Both probiotics similarly attenuated IS-induced inflammation in vitro and in patients when compared with the placebo [118].

The results of the main studies of probiotics in CKD are summarized in Table 4.

#### 5.2.3. Synbiotics

A synbiotic is an association of prebiotics and probiotics designed to enhance the survival and activity of the probiotics, with potentially greater beneficial effects than prebiotics or probiotics alone.

A randomized single-blind and placebo-controlled study on 58 hemodialysis patients compared a 7-week treatment with a synbiotic (extruded sorghum plus unfermented probiotic milk) with a control group (extruded corn plus pasteurized milk) and showed that the synbiotic group had decreased serum p-CS, IS and urea concentration, higher fecal butyric acid and lower intestinal pH compared to baseline and control group [119].

A prospective, non-randomized, placebo controlled trial on 50 non-dialysis CKD stage IV–V patients (matched for age, sex and primary disease) demonstrated that a 10-month synbiotic supplementation (*Lactobacillus acidophilus La-14* + fructooligosaccharides) determined a trend toward decreased serum IS levels without any significant effects on renal function and inflammatory (IL6) or oxidative stress markers [120].

#### 5.2.4. Postbiotics

A postbiotic is defined as a “preparation of inactivated, inanimate microorganisms and/or their components with the ability to confer a health benefit to the host” [121]. Postbiotics are inactivated bacteria or lysates of bacterial strains which include cellular fragments, such as cell wall components and pili, whereas by definition they should not encompass purified metabolites lacking cellular biomass such as SCFA (although these microbial metabolites might play an essential role in postbiotic preparations) [122].

Potential advantages of postbiotics compared to probiotics lie in their stability, even for several years at room temperature, and better safety profile due to inability to replicate (no risk of bacteremia). Mechanisms of action of postbiotics include modulation of resident microbiome and of microbiome-derived metabolites (increased levels of SCFA and vitamins), strengthening of the intestinal epithelial barrier, modulation of systemic or local immune responses, and modulation of systemic metabolic responses through the nervous system [123].

Preclinical studies have provided initial evidence that postbiotics may play a role in reducing inflammaging in CKD [123]. In a model of aged or adult mice, treatment with probiotics or probiotics and postbiotics mix (*Lactobacillus* and *Bifidobacterium* strains and their postbiotic antioxidant compounds) decreased oxidative stress markers in the kidneys [124].

The probiotic and postbiotics of four *Lactobacillus* and two *Bifidobacterium* strains were selected to inhibit Dextran sulfate sodium-induced colitis. The postbiotics were more effective than probiotics in preventing colitis and had a more pronounced effect in upregulating autophagy-related genes in the kidney. These results suggest that probiotics and postbiotics may reduce gut and kidney inflammation and modulate the gut–kidney axis by triggering the autophagy signaling pathway [125].

A lipolytic postbiotic derived from sonicated *Lactobacillus paracasei* (isolated from Egyptian cheese) was administered to a murine model of high-fat diet-induced kidney injury and compared to atorvastatin treatment or placebo. The postbiotic contained proteases, lipases and several antioxidant enzymes and displayed antibacterial activity against pathogenic bacteria. In postbiotic-treated rats, serum creatinine, uric acid and urea levels remained normal and were lower than in high-fat diet rats with or without atorvastatin treatment, suggesting a nephroprotective effect [126].

Interestingly, gut microbiota and oral microbiota are connected and deeply intertwined (“oral-gut axis,”) [127], and the oral microbiome of CKD patients could be colonized by enterobacteria in the periodontal pockets, favoring chronic systemic inflammation [128]. Beneficial modulation of both systems by postbiotics and synbiotics could represent a new possible adjuvant therapy in the management of both oral diseases (oral infections, periodontitis and uremic stomatitis) and CKD-related chronic inflammation [129].

#### 5.2.5. Meta-Analysis of Use of Biotics in CKD

Several meta-analyses have been published over the last decade in an attempt to clarify the role of prebiotics, probiotics and synbiotics on CKD [130,131,132,133,134] (Table 5). Although probiotics appear to reduce circulating levels of IS and p-CS and blunt chronic inflammation and oxidative stress in several studies, overall results are not conclusive.

The meta-analysis including the highest number of patients [133] concluded that very few studies adequately test biotic supplementation for key outcomes such as progression of CKD, cardiovascular events and circulating levels of uremic toxins. Furthermore, it is not yet established which type of approach among prebiotics, probiotics, synbiotics or postbiotics is more effective and whether it actually improves management of CKD as compared to standard care. On the other hand, adverse events have been consistently reported as mild and uncommon.

It must be underlined that these meta-analyses have several limits, as they globally analyze the effect of different types of biotics, dosages and study lengths and are generally too short to reliably appreciate potential beneficial effects on outcome such as, for example, renal function. Further studies are needed on selected probiotic strains (based on in vitro and in vivo evidence) and with targeted combination of synbiotics to support their use as an anti-inflammatory therapy in CKD [136].

### 5.3. Fecal Microbiota Transplantation (FMT)

FMT is a promising novel approach to treat CKD and has been investigated mainly in animal models in the setting of DKD [137]. FMT from healthy donor controls substantially reduced podocyte insulin resistance and blunted albuminuria and glomerular injury in diabetic rats, through downregulation of the GPR43 expression [138]. Life-long and repeated FMT from young mice improved age-related processes including intestinal permeability and metabolic and inflammatory profiles of macrophages [62].

In a pre-clinical murine model of type 2 DM, obesity and DKD, FMT prevented body weight gain, blunted albuminuria and TNF-α levels within the ileum and ascending colon, and ameliorated insulin resistance [139].

Despite these encouraging preclinical results, the experience of FMT in humans is still limited to small heterogeneous studies (Table 6). In the only currently available RCT, a total of 28 patients were included, and 15 of them were randomized to receiving FMT. Most of them had CKD from DKD, with significant proteinuria. After a 6-month observation, more patients (53.8%) from the placebo group progressed to CKD than the FMT group (13.3%), and the latter maintained stable renal function regardless of CKD stage. Adverse events after FMT treatment were mild gastrointestinal symptoms, which were not more frequent than those reported in the placebo group. FMT corrected alterations observed at baseline, reducing abundance of *Firmicutes* and *Actinobacteria* and increasing *Bacteroidetes*, *Proteobacteria* and *Roseburia spp. Arteaga-Muller* [140].

Other small non-controlled studies have reported positive preliminary results in glomerulonephritis such as IgA nephropathy (IgAN), idiopathic membranous nephropathy (IMN) and focal segmental glomerulosclerosis (FSGS) (Table 6). All these patients had received multiple lines of immunosuppressive therapy and were considered resistant or intolerant to conventional treatments [137]. Of interest, lower biodiversity and altered composition of gut microbiota were reversed by FMT in IgAN, with a decrease in phylum Proteobacteria and an increase in the genus Prevotella [141].

## 6. Current Limits and Future Perspectives

Our understanding of the mechanisms of the gut–kidney axis is still hindered by several limits of currently available studies.

Mechanisms of action of different types of biotics remain unclear, although they generally exert an anti-inflammatory effect. Limited study durations and populations may have made them inadequate to demonstrate the actual impact of biotics on hard outcomes such as kidney function, proteinuria, progression of CKD and reduction of cardiovascular events. Additionally, the included studies varied widely in biotic types, strains and dosages. Heterogeneous design and lack of standardization of different interventions have so far prevented definite conclusions on their effectiveness in reducing inflammaging in CKD, despite several meta-analyses. In addition, the best combinations of synbiotics and the role of postbiotics are areas which deserve further investigations. As a consequence, attempts at modulating gut microbiota with biotics still do not aim at targeting specific pathways, and biotics are still considered as an adjunctive treatment in CKD, often within the setting of a broader dietary intervention.

However, key progress has begun in the setting of feline CKD, in which multi-omics analysis has shed light on mechanisms underlying the effectiveness of two probiotic strains and defined “high responders” on the basis of reductions in gut-derived uremic toxins and increases in SCFA; this subset was characterized by peculiar microbiome composition and metabolite shifts [144]. Performing similar studies in humans would help “personalize” gut microbiota modulation and could provide an effective tool to monitor the actual impact of these therapies in modulating key microbial pathways involved in inflammation such as those involved in production of gut-derived uremic toxins (tyrosine, tryptophan, and phenylalanine metabolism) and SCFAs (pyruvate, propanoate, and butanoate metabolism). This targeted approach may help improve effectiveness of biotics and their assessment.

Furthermore, specifically designed multibiotics appear promising. Employment of an ex vivo “Simulator of the Human Intestinal Microbial Ecosystem” and in silico data mining has allowed the design of a multi-biotic (SynCKD) selecting *probiotic Lactobacillus johnsonii NCC533*, a prebiotic (1% cellobiose) and a postbiotic (1% short and medium chain triglycerides C4-C8, as a source of butyrate). The probiotic strain lacks the capacity to produce gut-derived uremic toxins as it does not have genes for tryptophanase, tyrosinase and urease. SynCKD effectively reduced uremic toxins generation in vivo in two uremic rodent models, resulting in lower plasma levels of uremic toxins, and enhanced kidney function after 6–8 weeks of treatment [145].

The role of specific detrimental bacteria is another interesting aspect which deserves further investigation. For example, *Akkermansia Muciniphila*, an anaerobic bacterium colonizing the mucus layer of human and animal gut, has been associated with different aging-related diseases including vasculopathy, type 2 diabetes and CKD [146].

On the other hand, specific beneficial bacteria have been identified: administration of *Lactobacillus johnsonii,* whose abundance is inversely correlated with CKD progression, can attenuate kidney lesion by suppressing the aryl hydrocarbon receptor (AHR) signal in a rat model of CKD [147]; similarly, supplementation of *Lacticaseibacillus rhamnosus* strain L34 reduced uremic toxins and blunted inflammation in non-dialysis CKD patients [118].

It is possible that targeting specific bacteria may enhance the effectiveness of biotics in specific settings such as CKD.

In addition, an expanded focus including not only bacteria but also other microorganisms, such as fungi, archaea and viruses could provide a broader insight into the gut–kidney axis [85].

Probably, time is ripe to move beyond analysis of relative composition of gut microbiota and potential associations with plasma UT levels and markers of chronic inflammation. We currently need in-depth exploration of gut microbiota in CKD by confounder-controlled quantitative microbial profiling, shotgun metagenomics and in vitro simulations of interactions among microorganisms and between them and the host. This could enable a rational selection of targeted biotic strategies based on in vitro and in vivo evidence and lead to a personalized intervention in CKD [148].

An improved capacity to modulate gut microbiota may represent a novel approach to treat many CKD-related manifestations, from sarcopenia to cardiovascular calcifications. Correction of gut microbiota dysbiosis can potentially translate in a reduction of inflammaging and of cardiovascular risk in CKD population, characterized by a peculiar, accelerated progression of senescence and atherosclerosis [149]. Interestingly, circulating levels of gut-derived uremic toxins can serve as biomarkers of cardiovascular risk and therapeutic targets and may enable measurement of the impact of biotics on this aspect [36].

## 7. Conclusions

Intestinal dysbiosis has been increasingly recognized as a key driver of chronic inflammation and inflammaging in CKD, and alterations of gut microbiota have been associated with all main CKD-associated manifestations, from cardiovascular disease to sarcopenia and malnutrition. Treatment with different types or associations of biotics (prebiotics, probiotics, synbiotics and postbiotics), as well as FMT, have provided interesting results in terms of reduction of circulating levels of gut-derived uremic toxins, suggesting a potential beneficial impact on chronic inflammation, cardiovascular risk and CKD progression. Further studies are needed to effectively harness gut bacteria and metabolic pathways and to explore synergistic benefits between diet and different biotics, as this may provide novel tools to attenuate chronic inflammation and improve CKD management.

## Figures and Tables

**Table 1:** Physiological functions of gut microbiota.

Function	Comment	References
Metabolic	GM regulates lipid and glucose metabolism by modulating both insulin sensitivity and lipogenesis. It also produces SCFA, bile acids, branched amino acids, vitamins and TMAO. Dysbiosis has been associated with obesity, liver steatosis and type 2 diabetes.	[16,17,18,19,20]
Immunomodulatory	GM promotes Treg development through specific bacterial species (*Lactobacillus* and *Bacteroides genera*) and interacts with GALT, intestinal epithelial and dendritic cells. Dysbiosis has been associated with rheumatoid arthritis, inflammatory bowel disease (IBD), and allergies.	[21,22]
Neuromodulatory	GM regulates neurobehavioral aspects by producing SCFA and precursors of neurotransmitters (e.g., serotonin) and affecting vagus nerve activity. Dysbiosis has been associated with mood disorders, autism and neurological disorders such as Parkinson’s disease	[23,24]
Preservation of gut barrier integrity	SCFA preserve and strengthen gut barrier. Dysbiosis has been associated with leaky gut syndrome (LGS) and endotoxinemia	[25,26]

Abbreviations: SCFA short chain fatty acid; TMAO Trimethylamine N-oxide; GALT Gut-associated lymphoid tissue; GM Gut Microbiota;w IBD inflammatory bowel syndrome; LGS Leaky gut syndrome.

**Table 2:** Main gut-derived uremic toxins (GDUT) and their potential effects on mechanisms of inflammaging.

Toxins	Substrate	Effects	References
IS	Tryptophan	Inhibition of nitric oxide (NO) production; boost of oxidative stress and disruption of endothelial repair through activation of aryl hydrocarbon receptor (AHR); enhanced monocyte infiltration within the kidneys and macrophage induction; senescence of proximal tubular cells and interstitial fibrosis. Associated with vascular calcifications, ventricular septal thickness, heart failure, CKD progression.	[33,37,38]
p-CS	Tyrosine	Cardiomyocyte apoptosis, vascular calcifications, endothelial dysfunction, activation of coagulation, boost of oxidative stress	[39,40,41,42]
IAA	Tryptophan	Increased production of ROS and oxidative stress through activation of AHR; increased expression of tissue factor	[41,43]
TMAO	Choline, carnitine, betaine	Activation of RAS and consequent renal fibrosis, cholesterol metabolism disruption, endothelial dysfunction, platelet activation, foam cell formation and atherosclerotic plaque instability	[44,45,46,47]
PAG	Phenylalanine	Activation of thrombotic pathways, increased platelet responsiveness; associated with stroke	[48,49]

Abbreviations: AHR aryl hydrocarbon receptor; CKD Chronic kidney disease; IAA: indole acetic acid; IS: indoxylsulfate; NO nitric oxide PAG: phenylacetylglutamine; p-CS: pericresylsulfate; ROS reactive oxygen species TMAO: trimethylamine N-oxide.

**Table 3:** Main studies on prebiotics in patients with CKD.

Author (Year)	Type of Prebiotic	CKD Stage	Number of pts	F/U (mo)	Main Results	References
MB Sohn (2023)	Inulin	IV	15	15	Expanded saccharolytic metabolites and abundance of Bifidobacterium and Anaerostipes	[99]
M Calderon Juarez (2024)	Inulin	IV	76	6	Decreased BUN and uremic symptoms	[100]
Y Zhang (2024)	Resistant starch (RS)		355		Decreased IS and BUN and improved renal function	[101]
SA Headley (2025)	RS	IIIa-IV	68	4	Reduction in p-CS. decrease in microbial α-diversity and Bacteroides	[102]
R Poesen (2016)	Galacto-oligosaccharides (GOS)	IIIb-IV	40	4	Sequential treatment with arabinoxylan oligosaccharides and maltodextrin (and vice versa) had no effect on GDUTs and insulin resistance	[103]
K Meksawan (2016)	Fructo-oligosaccharides (FOS)	Vd	9		Relief in constipation in elderly peritoneal dialysis patients	[104]

Abbreviations: BUN blood urea nitrogen; CKD Chronic Kidney Disease; FOS Fructo oligosaccharides; GOS Galacto oligosaccharides; GDUT gut-derived uremic toxins; IS Indoxyl sulfate; p-CS p-cresol sulfate; RS Resistant starch.

**Table 4:** Main studies on probiotics in patients with CKD.

Author (Year)	Type of Probiotic	Type of Nephropathy	Number of pts	F/U (mo)	Main Results	References
Miraghaja M. (2017)	probiotic soy milk	DKD	n = 48	2	Reduced oxidative stress as compared to controls.	[115]
Zhu H (2021)	*Lactobacillus Casei Zhang*	NA	n = 62	10	Reduced level of ACR as compared to controls. Increased expression of enzymes involved in NAD synthesis and of PGC1α, propionate and acetate synthesis in the kidney. It may reduce CKD progression in CKD 3–5 stages and risk of AKI-CKD transition.	[116]
De Mauri A (2022)	*Bifidobacterium Longum* and *Lactobacillus Reuteri*	Mainly nephrosclerosis and DKD	n = 60	5	Trend in reduction of GDUT and significant reduction of antihypertensive and loop diuretics in the group on probiotic	[102]
Leelahavanichkul A. (2025)	*Lacticaseibacillus rhamnosus* (L34) and *L rhamnosus GG* (LGG)	Mainly DKD and chronic GN	n = 75	1	L34 reduced gut-derived uremic toxins, systemic inflammation and gut permeability defect. LGG has comparable effect on IS-induced inflammation.	[118]

Abbreviations: ACR Albumin/creatinine ratio; AKI acute kidney injury; CKD chronic kidney disease; DKD Diabetic kidney disease; GDUT: gut-derived uremic toxins; GN glomerulonephritis; LGG Lacticaseibacillus rhamnosus GG; NA Not avaible; NAD nicotinamide; PGC1α Peroxisome proliferator-activated receptor gamma coactivator 1-alpha.

**Table 5:** Meta-analyses of effects of prebiotics, probiotics and synbiotics in patients with CKD.

Author (Year)	Number of pts (Studies)	CKD Stage	Intervention Duration	Main Results	References
Mc Farlane C (2019)	645 (16)	3 to 5	1–24 wks	Small or no difference in serum urea, IS and p-C; increased Bifidobacterium abundance. Limited evidence to support use of biotics in CKD.	[130]
Bakhtiary M (2021)	584 (14)	n.a.	4–12 wks	Reduction in total cholesterol, fasting blood glucose, insulin resistance and high-sensitivity CRP; increase in total anti-oxidant capacity	[135]
Liu J (2022)	842 (33)	3 to 5	4–24 wks	Improved total antioxidative capacity and reduced IL6, p-CS and IS in dialysis-dependent patients. No effect on GFR.	[131]
Chen C (2023)	780 (18)	3 to 5 (including dialysis)	4–12 wks	Reduced C-reactive protein, IL 6, and IS (not p-CS) and increased HDL-cholesterol in dialysis patients.	[132]
Cooper TE (2023)	2266 (45)	1 to 5 (including dialysis and renal transplanted patients).	6–12 wks	Uncertain effectiveness of synbiotics, prebiotics, or probiotics in improving patient outcomes in CKD compared to standard of care. Uncertainty on whether prebiotics, probiotics and synbiotics differ in effectiveness. Mild and uncommon adverse events.	[133]
Zhang Y (2024)	355 (645)	3 to 5	4–12 wks	Resistant starch dietary intervention can reduce IS (not p-CS) and urea nitrogen levels. No reduction in IL6 and TNFα.	[101]
Cedillo-Flores R (2025)	331 (20)	3 to 5	8–144 wks	Reduced levels of free and total IS and p-CS.	[134]

Abbreviations: CKD Chronic kidney disease; CRP C reactive protein; GFR glomerular filtration rate; HDL High density lipoprotein; IL-6 Interleukin 6.; IS Indoxyl sulfate; p-CS p-cresol sulfate; TNFα Tumoral necrosis factor α

**Table 6:** Studies on FMT in patients with CKD from different etiologies.

Author (Year)	Type of Nephropathy	Number of pts	F/U (mo)	Main Results	References
Arteaga-Muller G Y (2024)	DKD	n = 28	6	Reduced CKD progression	[140]
Zhao J (2021)	IgAN	n = 2	6	Reduction of proteinuria, increased serum albumin and stable renal function	[141]
Zhou G (2021)	IMN	n = 1	6	Reduction of proteinuria and anti-PLA2R antibodies, increased serum albumin.	[142]
Zhi W (2022)	FSGS	n = 1	12	Reduction of proteinuria and successful steroid tapering in a steroid-dependent form.	[143]

Abbreviations: antiPLA2R antibodies anti phospolipase A2 receptor; CKD Chronic kidney disease; DKD Diabetic kidney disease; FSGS Focal segmental glomerulosclerosis; IgAN Immunoglobulin A nephropathy.

## Data Availability

No new data were created or analyzed in this study.

## Abbreviations

The following abbreviations are used in this manuscript:

ACR	Albumine/creatinine ratio
AHR	Aryl hydrocarbon receptor
AXOS	Arabinoxylan oligosaccharide
BUN	Blood urea nitrogen
CRP	C reactive protein
DC	Dendritic cell
DKD	Diabetic kidney disease
ESRD	End stage renal disease
FMT	Fecal microbiota transplantation
Foxp3^+^ Tregs	Foxp3^+^ regulatory T cells
GALT	Gut-associated lymphoid tissue
GDUT	Gut-derived uremic toxins
CKD	Chronic kidney disease
GM	Gut microbiota
GNRI	Geriatric Nutritional Risk Index
GOS	α-galacto-oligosaccharides
FOS	β-fructooligosaccharides
HD	Hemodialysis
IAA	Indole acetic acid
IBD	Inflammatory bowel disease
Il1 β	Interleukin-1β
IS	Indoxyl sulfate
ILC3	Group 3 innate lymphoid cell
LGS	Leaky gut syndrome
LPD	Low-protein diet
MAPK	Mitogen-activated protein kinase
MD	Mediterranean diet
mtDNA	Mitochondrial DNA
mTOR	Mammalian target of rapamycin
mtROS	Mitochondrial Reactive oxygen species
NAD	Nicotinamide
NF-κB	Nuclear factor kappa-B
NLRP3	NOD-like receptor family pyrin domain containing 3
NO	Nitric oxide
PAG	Phenylacetylglutamine
PBD	Plant-based diets
PBUT	Protein-bound uremic toxins
PD	Peritoneal dialysis
p-CS	p-cresyl sulfate
PGC1α	Peroxisome proliferator-activated receptor gamma coactivator 1-alpha
ROS	Reactive oxygen species
RS	Resistant starch
SGA	Subjective global assessment
SASP	Senescence-associated secretory phenotype
Tr1	Type 1 regulatory T cells
